# Does the thoracic fluid content reflect lung water and cardiac preload?

**DOI:** 10.1007/s10877-025-01335-6

**Published:** 2025-08-23

**Authors:** Daniela Rosalba, Rui Shi, Chiara Bruscagnin, Christopher Lai, Gaëlle Fouque, Julien Hagry, Rosanna Vaschetto, Jean-Louis Teboul, Xavier Monnet

**Affiliations:** 1 Service de Médecine Intensive-Réanimation, Groupe de Recherche Clinique CARMAS, AP-HP, Hôpital de Bicêtre, DMU CORREVE, Université Paris-Saclay, Inserm UMR S_999, FHU SEPSIS, Le Kremlin-Bicêtre, France; 2 Dipartimento di Medicina Traslazionale, Università del Piemonte Orientale, Novara, Italy; 3 Azienda Ospedaliero-Universitaria Maggiore della Carità, Novara, Italy

**Keywords:** Thoracic fluid content, Hemodynamic monitoring, Biompedance, Volume expansion, Cardiac preload, Extravascular lung water, Acute respiratory distress syndrome, Transpulmonary thermodilution

## Abstract

Whether the thoracic fluid content (TFC) estimated by bioimpedance actually reflects the thoracic water content and tracks its changes has not been tested yet. We compared TFC changes induced by volume expansion with those of well-established markers of cardiac preload including the global end-diastolic volume indexed (GEDVI) estimated by transpulmonary thermodilution (TPTD) and central venous pressure (CVP). We assessed the relationship between TFC and extravascular lung water indexed (EVLWI) estimated by TPTD in patients with acute respiratory distress syndrome (ARDS). In 42 patients with a TPTD device (PiCCO2), we measured GEDVI, EVLWI and CVP before and after a 500-mL fluid bolus (*Fluid group*). In 23 different patients with ARDS, we measured the day-to-day changes in TFC and EVLWI (*ARDS group*). In the *Fluid group* (42 measurements), TFC increased significantly during fluid infusion. There was no correlation between the fluid-induced changes in GEDVI or CVP and the fluid-induced changes in TFC (*p* = 0.71 and *p* = 0.09, respectively). In the *ARDS group*, 124 measurements were performed, representing 101 changes between timepoints. There was no correlation between the percent changes in GEDVI, EVLWI, the sum of GEDVI + EVLWI and the percent changes in TFC (*p* = 0.52, 0.40 and 0.55, respectively). Considering all 208 measurements performed, absolute values of EVLWI were correlated with absolute values of TFC (*r* = 0.21, *p* = 0.04). There was no correlation between the absolute values of GEDVI and the sum of GEDVI + EVLWI and those of TFC (*p* = 0.33 and 0.39, respectively). Still considering all measurements performed, the percentage of error of cardiac index measured by bioreactance compared to TPTD was 131%. In critically ill patients, TFC measured by bioimpedance does not follow the changes induced by a fluid bolus of well-known markers of cardiac preload. It also does not follow the changes in EVLWI observed in patients with ARDS. While absolute values of TFC are weakly correlated with those of EVLWI, it is determined neither by GEDVI, nor by the sum of both.

**Supplementary Information:**

The online version contains supplementary material available at 10.1007/s10877-025-01335-6.

## Background

Electrical bioimpedance and bioreactance are two techniques that can be used for hemodynamic monitoring. Both are based on the principle that the electrical conductivity and impedance of the thorax are influenced by its volume [1, 2]. The Starling system (Baxter, Deerfield, IL, USA) measures stroke volume (and then cardiac output) through bioreactance, which is the difference in phase between the electrical inward current sent to the thorax by the system and the outward current [2]. Through bioimpedance, which is the ratio of voltage of the inward and outward electrical currents, the device also measures the mean transthoracic electric impedance (Z0). From bioimpedance, it derives the thoracic fluid content (TFC), assuming that TFC = 1000 / Z0 [3]. The TFC is supposed to include the fluid in the lungs (extravascular lung water and pulmonary blood volume), in the large thoracic vessels and the cardiac cavities [2]. However, whether it is really the case in critically ill patients has not been investigated yet.

The first objective of our study was to investigate whether TFC behaves as a marker of cardiac preload by comparing its changes induced by volume expansion with those of well-established markers of cardiac preload, including the global end-diastolic volume indexed for body surface (GEDVI) estimated by transpulmonary thermodilution (TPTD). The second objective was to assess the relationship between TFC and extravascular lung water indexed for ideal body weight (EVLWI) estimated by TPTD during acute respiratory distress syndrome (ARDS). The third objective was to establish the determinants of TFC, among hemodynamic variables including EVLWI and GEDVI estimated by TPTD.

## Methods

This prospective, observational, one-center study was conducted in the intensive care unit (ICU) of a tertiary hospital. Our study was approved by the Comité pour la protection des personnes Ile-de-France VII (SC9-003). The data used in this analysis were originally collected as part of a previously published prospective study (Gavelli et al., Annals of Intensive Care 2021). The present analysis was conducted retrospectively and was subsequently registered on ClinicalTrials.gov (2018-A02825-50/NCT05676723).

### Consent to participate

In the study was obtained from the patients or their next of kin.

### Patients

The inclusion criteria were (i) hospitalization in the ICU, (ii) monitoring by a calibrated TPTD device (PiCCO2, Pulsion Medical Systems, Getinge, Feldkirchen, Germany), (iii) a planned volume expansion as decided by the attending physicians (*Fluid group*) or a diagnosis of ARDS according to the Berlin definition [4] (*ARDS group*). Exclusion criteria were (i) age < 18 years, (ii) pregnancy, (iii) presence of extracorporeal membrane oxygenation (ECMO) assistance at the time of inclusion, (iv) impossibility to paste bioreactance electrodes properly to the skin of the thorax, (v) large pleural effusions, (vi) in *Fluid group*, circulatory failure whose treatment could not be postponed for ≥ 5 min (time required for setting up the Starling system), (vii) changes in the catecholamines dose or in the ventilatory settings performed during fluid infusion and (viii) unavailability of correctly analyzable data extracted from the PiCCO2 or the Starling System. Non-inclusion criteria were (i) unavailability of the investigators and (ii) refusal to join the study by the patient or his next of kin.

### Transpulmonary thermodilution measurements

In all patients, a thermistor-tipped femoral artery catheter and a central venous catheter were already in place as part of the patient’s hemodynamic monitoring. After calibrating the PiCCO2 system, the following TPTD variables were collected at baseline: cardiac index (CI), GEDVI, and EVLWI [5]. The results obtained from three injections of 15-mL cold saline boluses were averaged [6].

### Measurements with the Starling system

Once the patient was included, a Starling device was set up by pasting four sensors on the skin surface of the thorax, two sensors above and two below the heart, as recommended by the constructor. The device was not removed until the end of the study. This device measures CI through bioreactance and TFC through bioimpedance. The CI values displayed on the screen result from a 24-sec moving average [7, 8].

### Other variables

Demographic and other hemodynamic parameters, including heart rate, arterial blood pressure, and central venous pressure (CVP), extracted from the PiCCO2 device were recorded. Therefore, the CVP value was averaged over several respiratory cycles. The dose of sedatives drugs and catecholamines were also collected.

### Design of the study

#### Fluid group

Immediately after inclusion, the Starling device was set up and automatically self-calibrated. A first set of measurements was collected, including TPTD-derived variables (CI, GEDVI, EVLWI), TFC and bioreactance-derived CI. Volume expansion was then performed, according to the decision of the clinicians in charge, by infusing 500 mL of normal saline intravenously over 10 to 15 min. As per protocol in the ICU where the study was conducted, before administering the fluid bolus, preload responsiveness was tested with a passive leg raising (PLR) test. Patients who experienced an increase in CI ≥ 10% were considered preload responders. Immediately after the end of fluid infusion, TPTD was performed again and a second set of TPTD- and Starling-derived measurements was collected as before.

#### ARDS group

Once inclusion performed and until the PiCCO2 device was removed or the patient was extubated, at each time a TPTD measurement was performed according to current care, TFC and TPTD-derived variables were collected once a day at the same time. When several TPTD measurements were performed in a day, only the first one was considered.

### Statistical analysis

Distribution of variables was assessed visually. Data are expressed as median (interquartile range) or n (%). Comparison of variables between time points of the study was assessed using the Wilcoxon test. Comparisons between different groups of patients was performed using the Mann-Whitney U test.

For the *Fluid* group the main analysis consisted in comparing the changes induced by volume expansion in GEDVI, CVP and the sum of GEDVI + EVLWI on the one side and in TFC on the other side. For this purpose, we calculated the Spearman correlation coefficient between simultaneous changes. For the *ARDS* group the analysis consisted in comparing the relative changes of EVLWI and TFC between two successive measurements. For determining the determinants of TFC, considering all the measurements performed in both groups of patients, we calculated the Spearman correlation coefficient between absolute values of GEDVI, EVLWI, the sum of GEDVI + EVLWI on the one side and of TFC on the other side. In addition, we planned to perform a multiple regression analysis, in which the variable to explain was TFC, and the explaining variables were variables for which the p value of the correlation with TFC was < 0.1, among GEDVI, EVLWI, and the sum GEDVI + EVLWI. Univariate regression analyses were performed using Spearman’s correlation coefficient to assess the relationship between TFC and other hemodynamic variables. Multivariate regression analysis was not performed because none of the univariate correlations showed a p-value < 0.1, which was the predefined threshold to include variables in the model.

To investigate whether the difference between TFC and the sum of GEDVI + EVLWI could be related to the same factors as those explaining the difference between CI measured by bioreactance and by TPTD, we calculated the coefficient of correlation between the difference in TFC and GEDVI + EVLWI on the one side, and the bias between CI measured by bioreactance and CI measured by TPTD on the other side.

We calculated the least significant change in TFC in the first 10 patients included in the study. In these patients, during a period of hemodynamic stability (no change in mean arterial pressure and heart rate ≥ 5% compared to baseline during the last 15 min), the values of TFC were collected every 12 s for 15 min. We calculated the coefficient of variation of TFC as being the standard deviation divided by the mean of the 75 measurements [6, 9]. The precision was calculated as being two times the coefficient of variation, and the least significant change as coefficient of variation x 1.96 x √2 [6, 9].

The comparison between absolute values of CI measured by bioreactance and by TPTD measured at different timepoints was performed by using the Bland-Altman analysis for repeated measurements. The percentage error was calculated as 2 × SD divided by the mean of CI measured by TPTD. The changes in CI measured by bioreactance and by TPTD observed in both groups (induced by volume expansion in the *Fluid group*, between two successive time points in the *ARDS group*) were assessed by four quadrant analyses (with an exclusion zone of 12% [6]). The ability of the fluid-induced changes in CI measured by bioreactance to detect an increase in CI measured by TPTD ≥ 15%, defining volume responsiveness [10], was assessed by a receiving operating characteristic (ROC) curve analysis. Sensitivity, specificity, positive and negative predictive values are expressed as median (95% confidence interval). Grey zones were calculated using the method defining three levels of response: positive, uncertain, and negative. Uncertain responses were defined using a two-step procedure. We first calculated the 95% CI of the Youden’s index resulting from a 1000 population bootstrap. Then, we determined cut-off values for a sensitivity < 90% or a specificity < 90% (diagnosis tolerance of 10%). The largest interval from these two steps was used to determine the grey zone.

As this study was primarily designed as a proof-of-concept analysis, no formal sample size calculation was performed prior to inclusion. The reported values and thresholds were used post-hoc to verify whether the number of observations was sufficient to explore the relationships of interest. A p value < 0.05 was considered statistically significant. All tests were two-sided. Statistical analysis was performed using Medcalc software (version 20.218) (bvba, Mariakerke, Belgium).

## Results

### Patient characteristics

We included 42 patients in the *Fluid group* and 23 other patients in the *ARDS group* from January to August 2022. The patients were not consecutive (Supplementary Fig. 1). No patient was excluded. Patient characteristics are shown in Table 1. They were included 2 (1–4) days after their admission to the ICU. At inclusion, all patients were mechanically ventilated and sedated with propofol and remifentanil. Septic shock was the main cause of acute circulatory failure in the *Fluid group* and the only one in the *ARDS group* (Table 1). Among the 23 patients with ARDS, 12 patients (52%) suffered from pneumonia attributed to severe acute respiratory syndrome Coronavirus 2019.

**Table 1:** Patient characteristics in the two study groups

	Fluid group (*n* = 42)	ARDS group (*n* = 23)
Age (years)	64 [55–71]	66 [58–72]
Male sex (n, %)	31 (76%)	21 (91%)
Body mass index (kg/m^2^)	27 [23–29]	26 [23–28]
SOFA at admission	10 [8–12]	10 [9–12]
SAPS II	50 [39–59]	44 [39–54]
Type of shock (n, %)		
Septic Cardiogenic Hypovolemic	36 (86) 2 (5) 5 (12)	23 (100) 0 (0) 0 (0)
Lactate at inclusion (mmol/L)	2.1 [1.6–2.8]	2.3 [1.7–2.9]
Acute respiratory distress syndrome (n, %)	23 (55)	23 (100)*
Atrial fibrillation (n, %)	5 (12)	2 (9)
ICU length of stay (days)	12 [7–19]	17 [11–28]
ICU mortality rate (%)	21 (50)	15 (65)
Ventilatory settings Tidal volume (mL/kg PBW) Respiratory rate (breaths/min) FiO_2_ (%) Positive end expiratory pressure (cmH_2_O) Plateau pressure (cmH_2_O)	5.4 [5.4–6.1] 25 [25–28] 60 [40–70] 12 [8–14] 25 [20–29]	5.6 [5.1–6.2] 25 [25–28] 60 [40–80] 12 [9–14] 27 [23–31]
Drugs and sedation (n, %) Propofol Remifentanil Neuromuscular blocking agents Norepinephrine Dobutamine	42 (100) 42 (100) 18 (43) 40 (95) 7 (17)	23 (100) 23 (100) 17 (74)* 23 (100) 1 (4)
Dose of norepinephrine (µg/kg/min) Dose of dobutamine (µg/kg/min)	0.59 [0.53–0.70] 3.50 [2.75–2.87]	0.71 [0.52–0.87] 3.00 [2.87–3.50]

Data are expressed as n(%) or median [IQR]

* *P* < 0.05 vs. *Fluid group*

FiO₂: fraction of inspired oxygen, ICU: intensive care unit, PBW: predicted body weight, SAPS: Simplified Acute Physiology Score II at admission, SOFA: Sequential Organ Failure Assessment score at admission

### Fluid group

Hemodynamic variables before and after fluid infusion in these 42 patients are shown in Table 2. Fluid was infused because of a low CI in 37 patients, and with the aim of decreasing the dose of vasopressors in 5 patients. The infusion of the fluid bolus led to an increase in CI ≥ 15% in 23 (55%) volume responders. Simultaneously, GEDVI and TFC significantly increased by 6 [0–13] and 3 [2, 3, 4]%, respectively, while EVLWI did not change significantly (Table 2).

**Table 2:** Changes in hemodynamic variables induced by volume expansion in volume responders and non-responders in the *Fluid group*

	Before volume expansion	After volume expansion
Heart rate (min^− 1^)		
Volume responders (*n* = 23)	94 [85–109]	91 [83–106]
Volume non-responders (*n* = 19)	99 [87–110]	95 [87–110]
Systolic arterial pressure (mmHg)		
Volume responders (*n* = 23)	111 [88–120]	124 [111–140]*
Volume non-responders (*n* = 19)	117 [103–137]	134 [116–148]*
Diastolic arterial pressure (mmHg)		
Volume responders (*n* = 23)	54 [46–60]	65 [54–70]*
Volume non-responders (*n* = 19)	55 [50–65]	59 [52–69]
Mean arterial pressure (mmHg)		
Volume responders (*n* = 23)	68 [60–78]	80 [73–90]*
Volume non-responders (*n* = 19)	75 [70–85]	87 [75–92]*
Pulse pressure (mmHg)		
Volume responders (*n* = 23)	56 [39–59]	60 [51–78]
Volume non-responders (*n* = 19)	61 [54–71]	73 [58–81]
Central venous pressure (mmHg)		
Volume responders (*n* = 23)	8 [8–9]	10|9–11]*
Volume non-responders (*n* = 19)	12 [8–14]	12 [9–15]
PiCCO2 cardiac index (L/min/m^2^)		
Volume responders (*n* = 23)	2.3 [1.8–2.7]	3.0 [2.7–3.6]*
Volume non-responders (*n* = 19)	2.7 [2.2–3.5]	2.9 [2.2–3.5]*
Starling cardiac index (L/min/m^2^)		
Volume responders (*n* = 23)	2.3 [2.0-2.8]	2.8 [2.4–3.6]*
Volume non-responders (*n* = 19)	3.2 [2.4–3.9]	3.1 [1.3–3.5]
PiCCO2 stroke volume (mL)		
Volume responders (*n* = 23)	39 [33–47]	55 [44–59]*
Volume non-responders (*n* = 19)	47 [38–56]	53 [40–61]*
GEDVI (mL/m^2^)		
Volume responders (*n* = 23)	617 [555–687]	666 [622–752]*
Volume non-responders (*n* = 19)	685 [527–728]	710 [582–755]*
EVLWI (mL/kg PBW)		
Volume responders (*n* = 23)	11 [9–15]	13 [8–14]
Volume non-responders (*n* = 19)	12 [11–13]	12 [10–13]
Thoracic fluid content (/kΩ)		
Volume responders (*n* = 23)	74 [57–85]	76 [58–88]*
Volume non-responders (*n = 19)*	86 [69–117]	91 [71–119]*

*N* = 42. Data are expressed as median [IQR]

* *P* < 0.05 vs. Before volume expansion EVLWI: extravascular lung water indexed for predicted body weight, GEDVI: global end-diastolic volume indexed for body surface area, PBW: predicted body weight

**Fig. 1:**
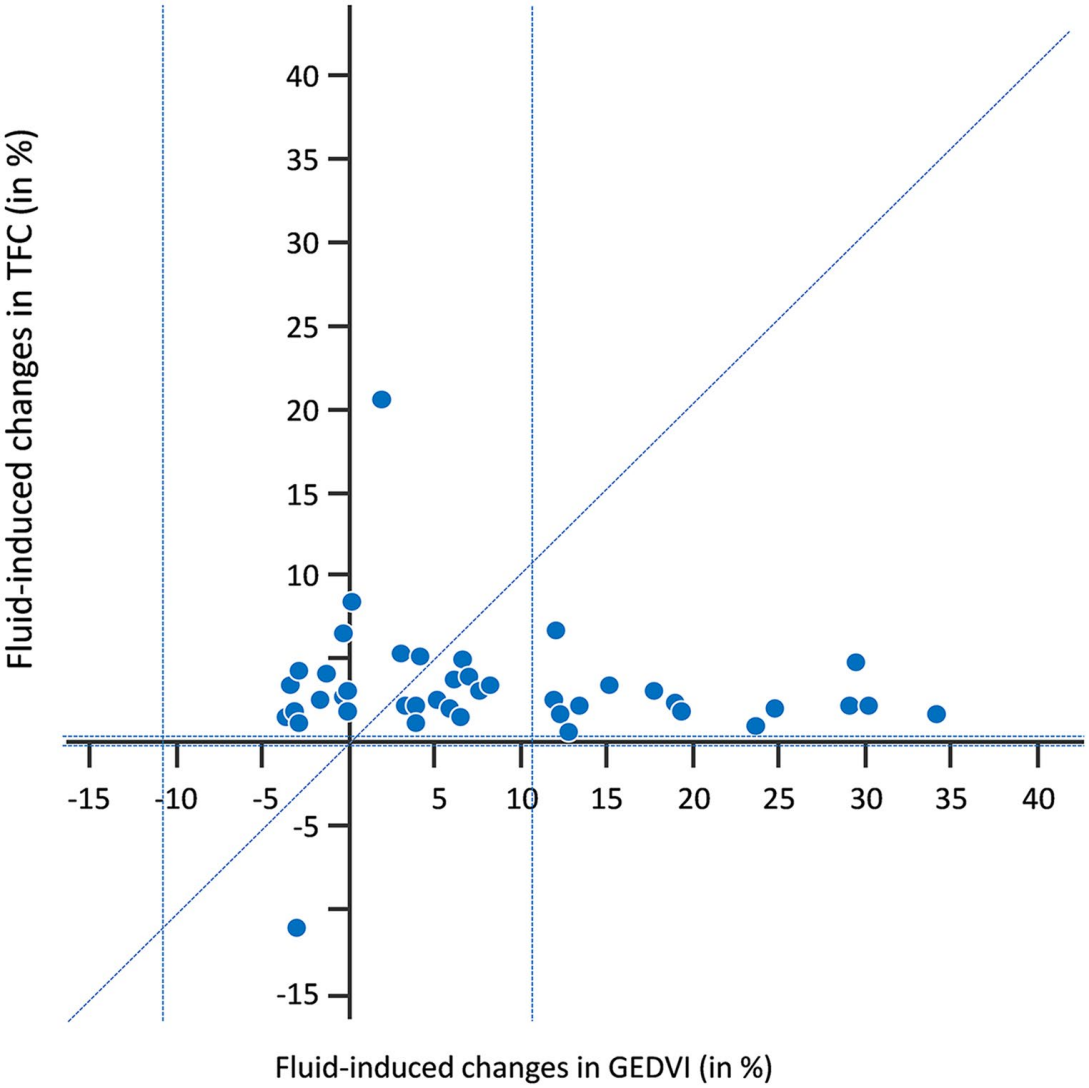
Correlation between the changes in thoracic fluid content (TFC) and in global end-diastolic volume indexed (GEDVI) induced by fluid infusion in the Fluid group

There was no correlation between the fluid-induced changes in GEDVI (*r* = -0.03, *p* = 0.71) (Fig. 1), in EWLVI (*r* = -0.09, *p* = 0.45), in the sum GEDVI + EVLWI (*r* = -0.02, *p* = 0.71) or in CVP (*r* = -0.44, *p* = 0.09) on the one side and the fluid-induced changes in TFC on the other.

### ARDS group

In the 23 patients of this group, 124 measurements were performed, representing 101 changes between timepoints. All measurements were performed in the supine position. On average, 10 (8–13) changes were measured in each patient. Their characteristics at inclusion are shown in Table 1, and their hemodynamic variables at inclusion in Supplementary Table 1.

Between two time points, in absolute value (i.e., non-negative values, without considering the direction of changes), GEDVI, EVLWI, the sum GEDVI + EVLWI and TFC changed by 14 [8–35]%, 15 [6–30]%, 11 [4–21]% and 11 [4, 5–22]%, respectively. There was no correlation between the percent changes in GEDVI (*r* = 0.04, *p* = 0.52), EVLWI (*r* = -0.07, *p* = 0.40), the sum of GEDVI + EVLWI (*r* = 0.03, *p* = 0.55) on the one side and the percentage changes in TFC on the other side. When considering only the first measurements performed in each patient (*n* = 23), there was also no correlation between changes in GEDVI (*r* =-0.10, *p* = 0.65), EVLWI (*r* = 0.05, *p* = 0.81), the sum of GEDVI + EVLWI (*r* = -0.10, *p* = 0.65) on the one side and the percent changes in TFC on the other side.

### Precision of the TFC measurements

The coefficient of variation of the TFC was 0.6, the precision was 0.1% and the least significant change was 0.2%.

### Accuracy of the estimation of cardiac output by bioreactance

Considering all measurements performed during the study (42 pairs in the *Fluid group*, 124 single measurements in the *ARDS group*), the bias between CI measured by bioreactance and by TPTD was 0.3 L/min/m^2^ and the limits of agreements were 2.0 and − 2.6 L/min/m^2^ (Supplementary Fig. 2). The percentage of error was 131%. The coefficient of correlation between the changes in CI measurements (42 induced by volume expansion in the *Fluid group*, 101 between two successive measurement points in the *ARDS group*) was 0.24 (*p* = 0.001).

In the *Fluid group*, an increase in CI measured by bioreactance ≥ 9% during fluid infusion detected a fluid-induced increase in CI measured by TPTD ≥ 15% with a sensitivity of 83 (63–95)% and a specificity of 89 (65–99)%, with an area under the ROC curve of 0.851 (95% IC: 0.707–0.942) (*p* < 0.001 vs. 0.5). In addition, a grey zone analysis based on the ROC curve identified a diagnostic uncertainty interval ranging from 5 to 13% change in CI measured by bioreactance, within which the discrimination of responders to fluid administration was inconclusive (Supplementary Fig. 3). The concordance rate outside the exclusion zone was 96% (Supplementary Fig. 4).

### Determinants of TFC

**Fig. 2:**
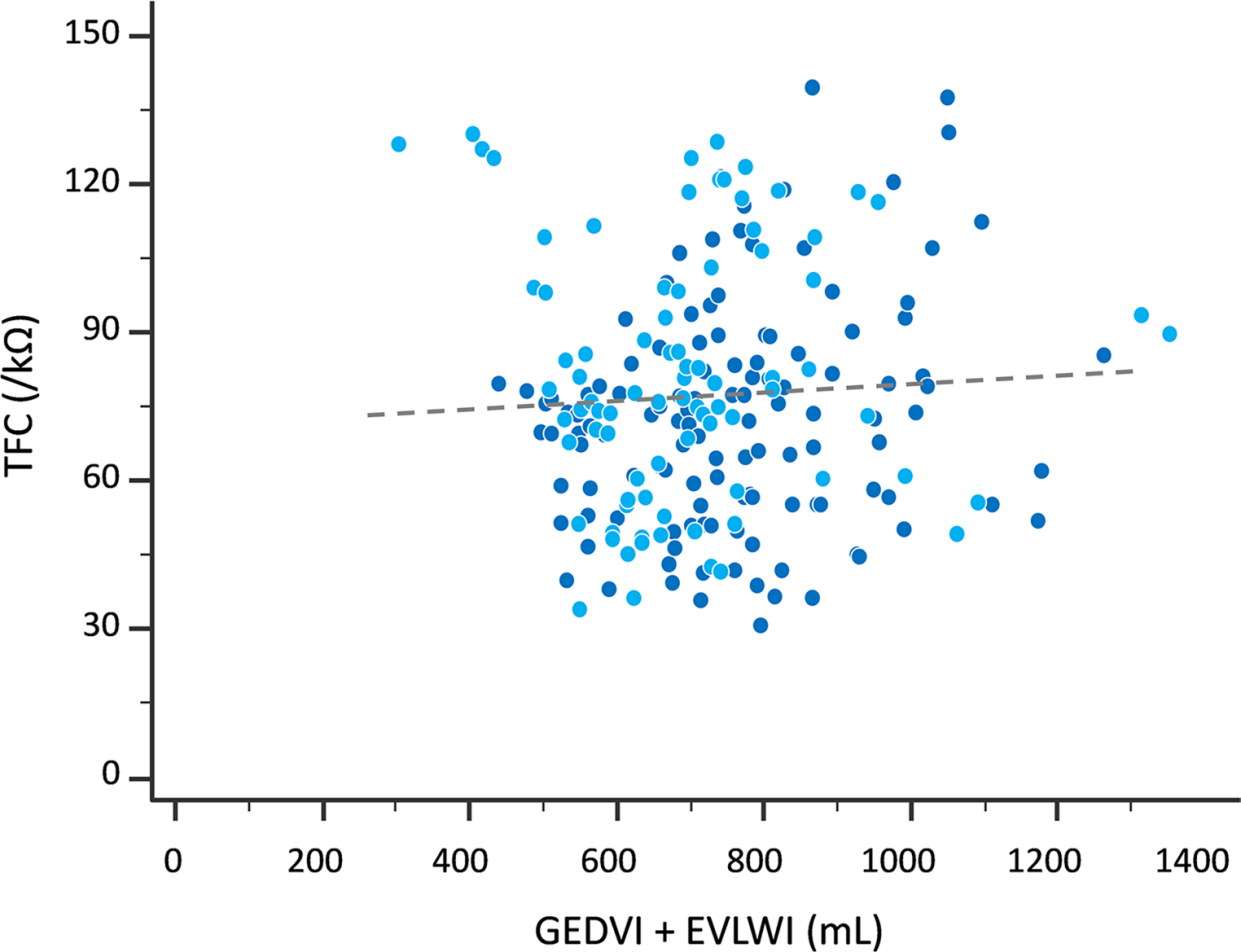
Correlation between the absolute values of thoracic fluid content (TFC) and the sum of global end-diastolic volume indexed (GEDVI) and extravascular lung water indexed (EVLWI) considering all measurements performed in the study. Patients of the Fluid group: dark blue (*n* = 84), patients of the EVLW group: light blue (*n* = 124). The dotted line indicates the regression line. *N* = 208, *p* = 0.39

Considering the 208 measurements performed in both groups (84 in the *Fluid group*, 124 in the *ARDS group*), there was no correlation between the absolute values of GEDVI (*r* = 0.07, *p* = 0.34) and the sum of GEDVI + EVLWI (*r* = 0.06, *p* = 0.40) (Fig. 2) on the one side and those of TFC on the other side. There was a significant correlation between absolute values of EVLWI and TFC (*r* = 0.21, *p* = 0.04).

There was no correlation between the bias in CI measured by bioreactance compared to TPTD on the one side and the difference between TFC and the sum GEDVI + EVLWI on the other side (*p* = 0.29).

## Discussion

This study, which was conducted in critically ill patients, shows that TFC measured by bioimpedance increased during the infusion of a fluid bolus. However, no significant correlation existed between the fluid-induced changes in TFC and those in GEDVI or CVP. In patients with ARDS, the changes in TFC were not correlated with the simultaneous changes in EVLWI over time. There was no correlation between absolute values of TFC and the sum of GEDVI + EVLWI, taking all measurements into account. The bias between TFC measured by bioimpedance and the sum of GEDVI + EVLWI was not correlated with the bias between CI measured by bioreactance and CI measured by TPTD.

Bioreactance is a non-invasive cardiac output measurement technique available today [11]. Alongside the measurement of cardiac output, the Starling system provides the measurement of TFC, estimated by bioimpedance (and not by bioreactance), supposed to estimate the volume of fluid contained in the thorax [3]. TFC has been demonstrated to predict cardiac events in patients with chronic heart failure [12, 13, 14]. It has also been used to assess the fluid status in children [15] or adults [16], especially during hemodialysis [17], pre-eclampsia [16], or weaning from mechanical ventilation [18]. It has been speculated that TFC could be used to guide fluid therapy especially in the peri-operative setting [19]. Since it should be partly composed of the fluid contained in the lung interstitium and the alveoli, it may also follow the evolution of ARDS severity. Nevertheless, as far as we know, whether TFC actually reflects the thoracic water content and tracks its changes has not been tested yet.

Our results all agree to invalidate this hypothesis. First, although TFC significantly increased during standardized volume expansion, these changes did not follow concurrent changes in either GEDVI or CVP, i.e., volumetric and barometric markers of cardiac preload, respectively. This is in agreement with a previous study showing the poor ability of TFC to estimate pulmonary artery occlusion pressure in decompensated chronic heart failure [20]. Second, in patients with ARDS, day-to-day lung water changes were not tracked by those of TFC. Considering all the measurements performed in the study, we found no correlation between the absolute values of TFC, and those of GEDVI, EVLWI or the sum of both. The degree of significance of these correlations prevented us from carrying out the multivariate regression analysis that we had planned to approach the determinants of the TFC.

An obvious limitation of our study may be that we compared TFC to GEDVI and EVLWI measured by TPTD, used as references. Indeed, there is no other method available at the bedside to estimate the different volumes of fluid contained in the thorax. Then, our results could be explained by the fact that the TFC includes other volumes than those estimated by the GEDVI and the EVLWI. The pulmonary blood volume, i.e., the volume contained in the pulmonary vessels [5], was not considered, nor was the volume of fluid contained in other thoracic spaces (pleural in particular) and tissues (muscles for example). Nevertheless, the fluid volume of the cardiac chambers (estimated by the GEDVI) and of the pulmonary tissue (estimated by the EVLWI) are so predominant in the thoracic total fluid content, that the absence of correlation of TFC with any of them casts doubt on its ability to estimate such volume in the entire thorax.

Another explanation for our results could also be that GEDVI and EVLWI do not provide a reliable measurement of the volumes that they are supposed to estimate. This may be the case for GEDVI which, even if it behaves as a preload marker [21], has been suspected of overestimating the real volume of the four cardiac chambers [22]. This is probably not the case for EVLWI. Indeed, several studies have shown that this index reliably approximates the actual volume of water contained in the interstitium and the pulmonary alveoli [23, 24].

Our study has several limitations besides those mentioned above. Firstly, it was carried out in critically ill patients, in whom the Starling system is not best indicated [25, 26]. Secondly, we were not able to explain the lack of correlation between the TFC on the one hand and the fluid volumes estimated by TPTD on the other hand. The precise method of TFC calculation is of course kept secret by the manufacturer. We also did not estimate whether these results were due to interferences between the device and the patient’s electrical environment, which were suspected to affect the reliability of bioimpedance [27]. Third, measurements of EVLWI after volume expansion were performed immediately at the end of the fluid bolus infusion, which may have minimized its changes because the increase in EVLWI could theoretically occur later. Finally, in the ARDS group, several changes were measured in the same patient. However, the analysis performed on the first measured change did not provide different results.

## Conclusion

In critically ill patients, the TFC measured by bioimpedance does not follow the changes in well-known markers of cardiac preload induced by a fluid bolus. It also does not follow the changes in EVLWI observed in patients with ARDS. It is determined neither by GEDVI, nor by EVLWI, nor by the sum of the two.

## Electronic supplementary material

Below is the link to the electronic supplementary material.

Supplementary Material 1

## Data Availability

All data supporting the findings of this study are available within the paper and its Supplementary Material.
