# Supplementary material for: Does the thoracic fluid content reflect lung water and cardiac preload?
Source: J Clin Monit Comput. 2025 Aug 23;39(5):1027–35. doi: 10.1007/s10877-025-01335-6 (PMC12474671; doi:10.1007/s10877-025-01335-6)
Supplement: Supplementary file 1 — Supplementary Material 1 [file 10877_2025_1335_MOESM1_ESM.docx]

Does the total fluid content reflect lung water and cardiac preload?

Daniela ROSALBA^(1,2,3)^, Rui SHI^(1)^, Chiara BRUSCAGNIN^(1)^, Christopher LAI^(1)^, Gaëlle FOUQUE^(1)^, Julien HAGRY^(1)^  , Rosanna VASCHETTO ^(2,3)^, Jean Louis TEBOUL^(1)^, Xavier MONNET^(1)^

Supplementary data

## Supplementary table 1

## Hemodynamic variables at inclusion in the *ARDS group*.

|  |  |
| --- | --- |
| Heart rate (min^−1^) | 85 [69-85] |
| Systolic arterial pressure (mmHg) | 134 [115-141] |
| Diastolic arterial pressure (mmHg) | 60 [55-67] |
| Mean arterial pressure (mmHg) | 84 [79-89] |
| Central venous pressure (mmHg) | 9 [6-11] |
| PiCCO2 cardiac index (L/min/m^2^) | 2.8 [1.8-3.1] |
| Starling cardiac index (L/min/m^2^) | 2.5 [1.9-3.5] |
| GEDVI (mL/m^2^) | 662 [547-722] |
| EVLWI (mL/kg PBW) | 16 [13-20] |
| Total fluid content (kOhm^-1^) | 72 [60-79] |

N=23. Data are expressed as median [IQR].

EVLWI: extravascular lung water indexed for predicted body weight, GEDVI: global end-diastolic volume indexed for body surface, PBW: predicted body weight.

Supplementary figure 1

Flowchart.

Supplementary figure 2

Comparison of cardiac index measured by bioreactance and by transpulmonary thermodilution according to the Bland-Altman analysis.

(CI_bio_: cardiac index measured by bioreactance, CI_TPTD_: cardiac index measured by transpulmonary thermodilution).

Supplementary figure 3

1. Receiver operating characteristic curve describing the ability of cardiac index measured by bioreactance to detect an increase in cardiac index measured by transpulmonary thermodilution ≥15% in the *Fluid group*.
2. Sensitivity and specificity of the changes in cardiac index induced by the PEEP test depending on the test result. The gray zone represents the uncertain zone with cut-off values with a sensitivity of <90% or a specificity of <90%.

(AUC: area under the curve).

A

B

Supplementary figure 4

Concordance analysis of the fluid-induced changes in cardiac index measured by bioreactance and of cardiac index measured by transpulmonary thermodilution in the *Fluid group.*

(CI: cardiac index, TPTD, transpulmonary thermodilution). N = 42
